# Divergent Hydraulic Safety Strategies in Three Co-occurring Anacardiaceae Tree Species in a Chinese Savanna

**DOI:** 10.3389/fpls.2016.02075

**Published:** 2017-01-18

**Authors:** Shu-Bin Zhang, Jiao-Lin Zhang, Kun-Fang Cao

**Affiliations:** ^1^Key Laboratory of Tropical Forest Ecology, Xishuangbanna Tropical Botanical Garden, Chinese Academy of SciencesMengla, China; ^2^Plant Ecophysiology and Evolution Group, Guangxi Key Laboratory of Forest Ecology and Conservation, College of Forestry, Guangxi UniversityNanning, China

**Keywords:** Chinese savanna, cavitation resistance, hydraulic safety margin, hydraulic-related traits, leaf phenology

## Abstract

Vulnerability segmentation, the condition under which plant leaves are more vulnerable to drought-induced cavitation than stems, may act as a “safety valve” to protect stems from hydraulic failure. Evergreen, winter-deciduous, and drought-deciduous tree species co-occur in tropical savannas, but there have been no direct studies on the role of vulnerability segmentation and stomatal regulation in maintaining hydraulic safety in trees with these three leaf phenologies. To this end, we selected three Anacardiaceae tree species co-occurring in a Chinese savanna, evergreen *Pistacia weinmanniifolia*, drought-deciduous *Terminthia paniculata*, and winter-deciduous *Lannea coromandelica*, to study inter-species differentiation in leaf and stem hydraulic safety. We found that the two deciduous species had significantly higher sapwood-specific hydraulic conductivity and leaf-specific hydraulic conductance than the evergreen species. Moreover, two deciduous species were more vulnerable to stem cavitation than the evergreen species, although both drought-deciduous species and evergreen species had drought-resistance leaves. The evergreen species maintained a wide hydraulic safety margin (HSM) in stems and leaves; which was achieved by embolism resistance of both stems and leaves and isohydric stomatal control. Both deciduous species had limited HSMs in stems and leaves, being isohydric in the winter-deciduous species and anisohydric in drought-deciduous species. The difference in water potential at 50% loss of hydraulic conductivity between the leaves and the terminal stems (P50_leaf−stem_) was positive in *P. weinmanniifolia* and *L. coromandelica*, whereas, *T. paniculata* exhibited a lack of vulnerability segmentation. In addition, differences in hydraulic architecture were found to be closely related to other structural traits, i.e., leaf mass per area, wood density, and sapwood anatomy. Overall, the winter-deciduous species exhibits a drought-avoidance strategy that maintains the hydraulic safety of the more carbon-costly stems by sacrificing cheaper and more vulnerable leaves, while the evergreen species exhibits a hydraulic strategy of drought tolerance with strong stomatal regulation. In contrast, the drought-deciduous species lacks vulnerability segmentation and sheds leaves at the expense of top shoots during peak drought. This study demonstrates that even sympatric tree species that differ in leaf phenology can exhibit divergent adaptive hydraulic safety strategies.

## Introduction

In tropical seasonal dry forests and savannas, variation in water availability puts a selective pressure on plants and drives ecological differentiation in hydraulic architecture and life history strategies (Markesteijn et al., 2011; Fu et al., 2012). Leaf phenology is a common type of ecological differentiation among coexisting tree species with contrasting water-use strategies (Holbrook et al., 1995; Choat et al., 2005). For instance, evergreen species are expected to be less vulnerable to drought-induced cavitation, whereas, deciduous species are drought-avoiding during dry periods (Holbrook et al., 1995; Choat et al., 2005; Ishida et al., 2010). Furthermore, deciduous species can also be classified into winter-deciduous and drought-deciduous depending on whether they shed leaves in early or in peak drought, respectively (Zhang et al., 2007). Regardless of leaf phenological type, tree growth and survival depend upon the maintenance of hydraulic safety during dry periods (Zimmermann, 1978; Markesteijn et al., 2011). The potential hydraulic safety strategies of tree species differing in leaf phenology remain poorly understood, however.

Hydraulic safety margins (HSMs) reflect the degree of hydraulic conservatism of a plant or a given organ (Meinzer et al., 2009; Bucci et al., 2013; Johnson et al., 2016). HSM has been most commonly calculated as the difference in water potential between the minimum value experienced in the field and the value at either 50% loss of xylem hydraulic conductivity (P50_stem_) or at 50% loss of leaf hydraulic conductance (P50_leaf_) (Meinzer et al., 2009). HSM has been used to estimate the water potential threshold leading to catastrophic hydraulic failure in leaves and stems (Meinzer et al., 2009; Choat et al., 2012; Liu et al., 2015). Plants have evolved adaptive strategies to keep water potential above the critical point at which drought-induced hydraulic dysfunction occurs (Delzon and Cochard, 2014). Because the branches are carbon-costly constructed and less phenotypically plastic than distal leaves, plants should protect basal organs by sacrificing distal portions (Johnson et al., 2016). However, some plants such as riparian cottonwoods coped with drought stress by branch die-back, an extreme mechanism of drought adaptation (Rood et al., 2000). Plants with different leaf habits may employ diverse strategies of drought tolerance (Choat et al., 2005; Zhang et al., 2007), and further studies are needed to better understand the relationship between leaf phenology and the different approaches to sustain hydraulic safety in plant organs.

Vulnerability segmentation between proximal and distal organs—between the leaves, which are more vulnerable, to drought-induced cavitation, and the terminal stems—is known to act as a “safety valve” to protect hydraulic pathways from dysfunction (Tyree et al., 1993; Liu et al., 2015; Zhu et al., 2016). The role of leaves as “safety valves” is evidenced by their hydraulic resistance that acts as a hydraulic bottleneck at the leaf level (Pivovaroff et al., 2014). Under midday water deficit or seasonal drought, the occurrence of xylem embolism in leaves sends a signal for stomatal closure to reduce water loss by transpiration (Brodribb and Holbrook, 2003a,b, 2004; Zhang et al., 2013). Stomatal control can thereby limit the decrease in water potential and maintain hydraulic safety under drought (Johnson et al., 2009; Zhang et al., 2013; Nolf et al., 2015).

Several studies have revealed that deciduousness is a successful strategy for surviving drought and avoiding hydraulic failure (Poorter and Markesteijn, 2008; Markesteijn et al., 2011). Leaf shedding considerably lowers water loss by transpiration and prevents catastrophic hydraulic failure during severe drought stress (Choat et al., 2005; Chen and Cao, 2015). More recently, Zhu et al. (2016) found that tree species from arid areas commonly exhibit vulnerability segmentation. Nevertheless, it requires further studies to determine whether vulnerability segmentation maintains the hydraulic safety of tree species differing in leaf phenology in arid ecosystems.

To a large extent, the differences in hydraulic efficiency and safety between evergreen and deciduous trees are closely related to leaf and stem structural characteristics (Choat et al., 2005; Chen et al., 2009; Fu et al., 2012). With respect to sapwood anatomy, the wider and longer vessels of deciduous species allow greater hydraulic efficiency but are more vulnerable to cavitation, whereas, the narrower and shorter vessels of evergreen species have higher cavitation resistance (Chen et al., 2009; Hacke and Jansen, 2009; Ishida et al., 2010). As conduit wall thickness and wood density increase, cavitation resistance is enhanced at the expense of hydraulic efficiency (Tyree et al., 1994; Lens et al., 2011). Consequently, tree species differing in leaf phenology must have co-evolved with structural traits that reflect a trade-off between hydraulic efficiency and safety.

Because of the rain shadow effect from the mountains in southwest China, the river valleys are characterized by a dry-hot climate with two distinct seasons: a rainy season and a dry season. These valleys host a valley-type savanna (Jin and Ou, 2000), in which evergreen, drought-deciduous, and winter-deciduous tree species co-occur (Zhang et al., 2007). The winter-deciduous species shed all leaves in the early dry season. The drought-deciduous species shed leaves in the late dry season, but the leaves do not shed completely except in extreme drought when all leaves are shed off. All three phenological types can acclimate to the local dry-hot environment, however, relatively little information is known about the differences in hydraulic safety in the leaves and stems among the three phenological types.

In the study, we investigated leaf and stem hydraulic properties as well as seasonal variations in water potential and stomatal conductance in three sympatric tree species of Anacardiaceae in a Chinese savanna, which have evergreen, drought-deciduous, and winter-deciduous leaf habits. The aims of our study were to identify the potential divergent strategies that maintain the hydraulic safety of the whole plant, and also to identify the hydraulic-related structural traits responsible for inter-species differences in hydraulic safety.

## Materials and methods

### Study site and plant species

This study was conducted at the Yuanjiang Savanna Ecosystem Research Station (YSERS, lat. 23°27′ N, long. 102°10′ E, elevation 481 m) of Xishuangbanna Tropical Botanical Garden, Chinese Academy of Sciences, located in Yuanjiang county, Yunnan province, southwestern China. The climate is characterized by two distinct seasons: a rainy season (May to October) and a dry season (November to next April). Based on YSERS meteorological data from 2012 to 2015, the mean annual temperature was 24.9°C, and the mean annual precipitation was 694.7 mm, with 80.4% of the precipitation falling during the rainy season (Supplementary Figure 1).

In this study, three sympatric species of Anacardinaceae *Pistacia weinmanniifolia, Terminthia paniculata*, and *Lannea coromandelica*, which exhibit evergreen, drought-deciduous, and winter-deciduous phenological types, respectively, were selected. These three tree species belong to the same family, therefore, the impact of phylogenetic differences is largely ruled out. YSERS established a 1 ha (100 × 100 m) long-term monitoring plot in 2011. In this plot, five mature individuals per species growing in full sunlight were selected. The upper canopy and sun-exposed branches with fully expanded and healthy leaves were used for the measurements. Information on these sample trees is summarized in Supplementary Table 1.

### Leaf and stem water potentials

Leaf water potential (Ψ_leaf_, MPa) was measured using a pressure chamber (PMS, Corvallis, OR, USA). Two healthy leaves were excised from each of five trees per species before dawn (6:00–7:00) to measure the predawn Ψ_leaf_ on continuously sunny days in the rainy season (July), the early dry season (November), and the late dry season (March). To estimate midday stem water potential (midday Ψ_stem_), the terminal branches were wrapped with plastic bags and aluminum foil in the evening before the measurement days, and the leaves were assumed to be in equilibrium with the xylem water potential of the terminal stems. The bagged and non-bagged leaves were collected at midday (12:00–14:00) on the same days and used to measure midday Ψ_stem_ and Ψ_leaf_, respectively. For *L. coromandelica* and *T. paniculata*, healthy leaves from the early and late dry season, respectively, were used to measure Ψ_leaf_ before leaf shedding occurred. According to the soil water monitoring data in the plot at YSERS, the water potential in topsoil was −0.29, −1.04, and −1.80 MPa in July, November, and March, respectively, when the seasonal samples were collected.

### Stem hydraulic conductivity and vulnerability curves

During the rainy season of 2014, terminal branches were harvested from five individuals of each species before dawn. These samples were sealed with moist towels and transported immediately in a cool box to a nearby laboratory for further processing.

Stem hydraulic conductivity was measured using the method described by Sperry and Tyree (1988). The terminal branches were re-cut under distilled water and both ends of the stem segment were shaved with a sharp razor blade. In the laboratory (20–25°C), stem segments were flushed at a pressure of 0.1 MPa with potassium chloride (KCl) solution for at least 20 min to remove air embolisms. The direction of the injection flow was always from the base to the top of the stems. The stem segment was connected to a hydraulic conductivity apparatus (Sperry et al., 1988). An elevated water reservoir generated a gravity-induced hydrostatic pressure (7.3 kPa), which drove the flushing solution (0.1 mol L^−1^ KCl solution) through the segments. One end of the segment was connected to this flushing solution and another end was connected to a pipette for measuring the water flow rate through the segment. Hydraulic conductivity per unit pressure gradient (*K*_h_, kg m s^−1^ MPa^−1^) is equal to the ratio between the water flux through an excised stem segment and the pressure gradient causing the flow. Afterwards, the sapwood of the segment was flushed with a methyl blue solution and the cross sapwood area (A_S_, mm^2^) was calculated as the mean value of the cross section of the two ends of the stem segment. Total distal leaf area (A_L_, m^−2^) for every terminal stem was determined with a Li-3000A leaf area meter (Li-Cor, Lincoln, NE, USA), and the dry mass of the leaves was determined after drying in an oven at 70°C for 48 h. Leaf mass per area (LMA) was calculated as the ratio of leaf dry mass to A_L_. Sapwood-specific hydraulic conductance (*K*_S_, kg m^−1^ s^−1^ MPa^−1^) is equal to *K*_h_ divided by A_S_. Specific leaf hydraulic conductivity (*K*_L_, kg m^−1^ s^−1^ MPa^−1^) was calculated as the ratio of *K*_h_ to A_L_. In addition, the Huber value (HV, mm^2^ m^−2^) was calculated, indicating the amount of cross sapwood area (A_S_) per unit of distal leaf area (A_L_).

The maximum vessel length (MVL) was determined using the air injection method (Brodribb and Field, 2000). Stem segments longer than the MVL were used to determine stem hydraulic conductivity. Stem vulnerability curves were measured by the air injection method (Cochard et al., 1992). The above segments were placed inside a pressure chamber (PMS, Corvallis, OR, USA) with both ends protruding. The proximal ends were connected to the measuring equipment, and maximum hydraulic conductivity was measured. We raised the pressure inside the chamber to 0.5 MPa and maintained it for at least 10 min, then reduced it to a basal level of 0.01 MPa. We waited for more than 10 min to allow the system to equilibrate, and then we repeated the process, raising the injection pressure by 0.5 or 1.0 MPa each time until more than 80% of *K*_S_ was lost. The residual pressure inside the chamber was maintained to ensure that no refilling could occur during measurements. We fitted the vulnerability curve using the following equation (Pammenter and Vander Willigen, 1998):

(1) $$\begin{array}{l} \text{PLC}=\frac{100}{1+\exp\;\left[\text{a}\left(P-\text{b}\right)\right]} \end{array}$$

where PLC (%) is the percentage loss in stem hydraulic conductivity, *P* is the xylem water potential, and the parameters a and b are the maximum slope of the curve and xylem water potential at 50% loss of stem hydraulic conductivity (P50_stem_), respectively.

### Leaf vulnerability curves

Leaf vulnerability curves were determined using the method described by Franks (2006). Leaves were sampled before dawn and held under water to rehydrate, and then the leaves with different water potentials were used to determine the leaf vulnerability curves. The chamber pressure was maintained at the leaf water potential (Ψ_1_) and allowed to equilibrate for about 2 min. The chamber pressure was then rapidly increased to Ψ_2_ (Ψ_2_ was 0.2–0.5 MPa more than Ψ_1_) and the sap was collected within 10 s (*WM*, g) and weighed on a balance (accurately measured to 0.0001 g). The leaf was scanned with a scanner (HP Scanjet 3110 Photo Scanner, Hewlett-Packard, USA) and leaf area (LA, cm^2^) was calculated using ImageJ software (http://imagej.nih.gov/ij/). *K*_leaf_ was determined as follows:

(2) $$\begin{array}{l} K_{\text{leaf}}\;=\;\frac{WM}{10^{*}M^{*}{\left(\psi_{\text{2}}\;-\;\psi_{\text{1}}\right)}^{*}LA} \end{array}$$

where the parameter M is the molar mass of water (18 g mol^−1^). The relationships between *K*_leaf_ and Ψ_leaf_ were fitted to a sigmoidal curve, and the leaf water potential at 50% loss of *K*_leaf_ (P50_leaf_) was derived from to the fitted equation.

### Hydraulic safety margins

In this study, two types of HSMs were calculated and used to compare inter-species differences among leaf phenologies. First, the differences in the minimum Ψ_leaf_ or Ψ_stem_ values and the values at which 50% of the hydraulic conductance was lost (P50_leaf_ or P50_stem_) were defined as the HSMs in the leaves and stems, respectively (Bucci et al., 2013): HSM_(*leaf*)_ = minimum Ψ_leaf_ − P50_leaf_; HSM_(stem)_ = minimum Ψ_stem_ − P50_stem_. The minimum values of Ψ_leaf_ and Ψ_stem_ occurred at midday during the early dry season for *L. coromandelica*, and during the late dry season for *P. weinmanniifolia* and *T. paniculata* (Table 1). In order to reflect the intrinsic drought tolerance abilities under seasonal drought of plants in the field, we used the minimum values of Ψ_leaf_ and Ψ_stem_ in the late dry season for *P. weinmanniifolia* and *T. paniculata*, and the minimum values in the early dry season for *L. coromandelica*.

**Table 1:** **Seasonal variation in water potential and stomatal conductance in three Anacardiaceae tree species**.

**Species**		**Rainy season**	**Early dry season**	**Late dry season**
*Pistacia weinmanniifolia*	Predawn Ψ_leaf_	−0.48 ± 0.06a	−0.63 ± 0.02a	−1.20 ± 0.07b
	Midday Ψ_leaf_	−1.21 ± 0.07a	−1.28 ± 0.07a	−1.33 ± 0.04a
	Midday Ψ_stem_	−0.54 ± 0.05a	−0.60 ± 0.06a	−0.88 ± 0.05b
	*g*_s_	0.16 ± 0.02a	0.13 ± 0.01a	0.04 ± 0.01b
*Terminthia paniculata*	Predawn Ψ_leaf_	−0.53 ± 0.03a	−0.73 ± 0.03b	−1.17 ± 0.04*c*
	Midday Ψ_leaf_	−1.12 ± 0.06a	−1.45 ± 0.09a	−2.70 ± 0.18b
	Midday Ψ_stem_	−0.53 ± 0.03a	−0.83 ± 0.05a	−2.32 ± 0.07b
	*g*_s_	0.25 ± 0.01a	0.22 ± 0.01a	0.14 ± 0.02b
*Lannea coromandelica*	Predawn Ψ_leaf_	−0.43 ± 0.02a	−0.98 ± 0.07b	–
	Midday Ψ_leaf_	−1.23 ± 0.05a	−1.30 ± 0.03a	–
	Midday Ψ_stem_	−0.52 ± 0.07a	−0.75 ± 0.05b	–
	*g*_s_	0.25 ± 0.01a	0.05 ± 0.01b	–

*Values are means ± SE (n = 6). Different letters indicate significant seasonal differences within a species at P < 0.05. Ψ_leaf_, leaf water potential (MPa); Ψ_stem_, stem water potential (MPa); g_s_, stomatal conductance (mol m^−2^ s^−1^)*.

Second, considering that leaves are more vulnerable to cavitation than stems, we quantified the differences in water potential at 50% loss of hydraulic conductance between the leaves and the terminal stems (P50_leaf−stem_) (Johnson et al., 2012).

### Stomatal conductance

Stomatal conductance (g_s_) was determined after photosynthetic induction for 20 min under a reference CO_2_ concentration of 400 μmol mol^−1^ and a photosynthetic photon flux density (PPFD) of 1200 μmol m^−2^ s^−1^. These measurements were made from five sun-exposed individuals of each species using a portable gas analysis system (LI-6400, Li-Cor, Lincoln, NE, USA) between 9:00–11:00 on sunny days in the three seasons. The tested trees were the same ones used for the hydraulic measurements.

### Wood density and sapwood anatomy

The branch segments used for the hydraulic conductivity measurements were also used to measure sapwood density (WD, g cm^−3^) and xylem anatomy. For sapwood density, 3-cm-long portions were used. The volume of fresh sapwood was measured using the water displacement method after removing the bark and pith material. The sapwood sample was dried in an oven at 70°C for 48 h and then the dry mass was determined. WD was calculated as the ratio of the dry mass to the volume of the fresh sapwood.

For anatomical measurements, 5-cm-long segments were sampled from the stems used for the hydraulic conductivity measurements and these segments were stored in 1:1 (v/v) ethanol and glycerol solutions for at least 3 months. Transverse sections about 15–30 μm thick were made with a microtome and vessel diameters were measured under a DM2500 light microscope (Leica Inc., Bensheim, Germany) connected to a computer. The digital images were then examined with ImageJ software (http://rsbweb.nih.gov/ij/). At least 30 vessels for each species were chosen from the latest-growth sapwood. Vessel diameter (D_h_, μm) was calculated as (D^4^/N)^1/4^, where N was the number of vessels (Tyree and Zimmermann, 2002). Vessel wall thickness (VWT, μm) was half of the inter-vessel wall thickness. Moreover, vessel density (VN, no. mm^−2^) was measured as the number of vessels per square millimeter of sapwood area. In sapwood anatomy, vessels that group together are defined as a vessel group, the members of which are linked by inter-vessel pits; a solitary vessel was still counted as one vessel group (Lens et al., 2011). The vessel grouping index (VGI) was calculated as the ratio of the number of vessels to the number of vessel groups in certain digital images (Wheeler et al., 2005; Lens et al., 2011).

### Data analysis

Statistical analysis was performed with SPSS 16.0 software (SPSS, Chicago, IL, USA). The differences in hydraulic traits among the three species and the seasonal differences within the species (*T*. *paniculata* and *P. weinmanniifolia*) were assessed by one-way analysis of variance (ANOVA) with least significant difference (*LSD*) multiple comparisons at a significance level of *P* < 0.05. In addition, the seasonal difference in *L. coromandelica* was assessed by independent *t*-tests at *P* < 0.05. The figures were plotted with SigmaPlot software version 10.0.

## Results

### Seasonal variation in plant water potential and stomatal conductance

Predawn Ψ_leaf_ decreased slightly in the early dry season and significantly in the late dry season for *P. weinmanniifolia* (Table 1, *P* < 0.001). The same trend was found in midday Ψ_stem_. *P. weinmanniifolia* maintained a constant midday Ψ_leaf_ irrespective of the season. The values of *g*_s_ in the early and late dry season decreased to 80.8% (*P* = 0.103) and 26.5% (*P* < 0.01), respectively, of the value in the rainy season. Significant seasonal differences were found in predawn Ψ_leaf_ in *T. paniculata* (*P* < 0.001). By peak drought, the values of midday Ψ_stem_ and midday Ψ_leaf_ in that species significantly decreased to −2.32 MPa (*P* < 0.01) and −2.70 MPa (*P* < 0.001), respectively. The values of *g*_s_ in the early and late dry season decreased to 88.0% (*P* = 0.135) and 56.8% (*P* < 0.001) of the value in the rainy season, respectively. By the early dry season, predawn Ψ_leaf_ and midday Ψ_stem_ significantly decreased compared with the values in the rainy season for *L. coromandelica* (*P* < 0.001 and *P* < 0.05, respectively), but no significant seasonal difference was found in midday Ψ_leaf_. Correspondingly, *g*_s_ decreased to 18.2% of the value in the rainy season (*P* < 0.001).

### Hydraulic conductivity and vulnerability in leaves and stems

**Figure 1:**
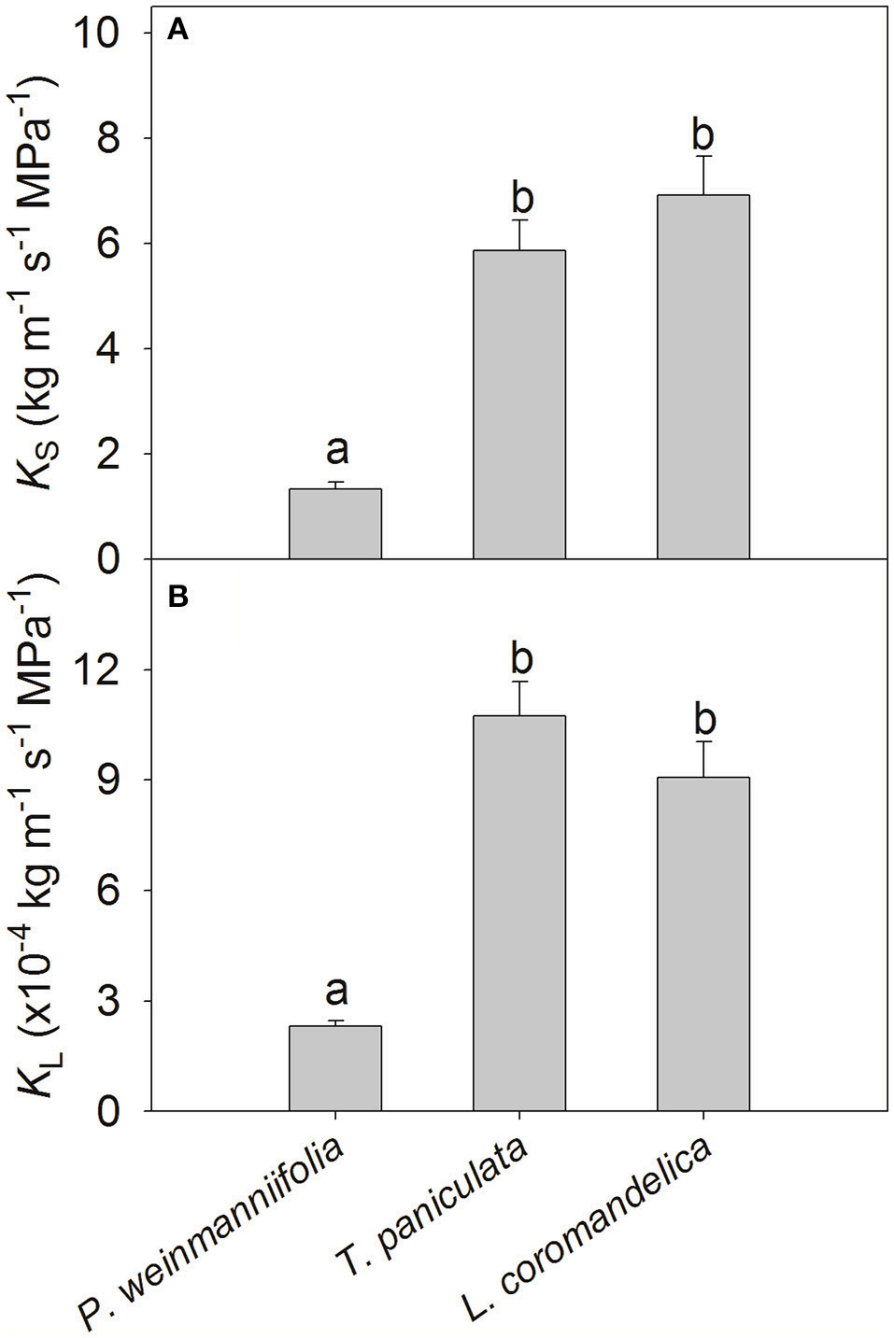
**Sapwood-specific hydraulic conductivity (***K***_**S**_; A)** and leaf-specific hydraulic conductivity (*K*_L_; **B**) of three Anacardiaceae tree species. Different letters above the bars indicate significant differences by the LSD *post-hoc* test following a one-way ANOVA at *P* < 0.05. Values are means ± SE (*n* = 5).

*T. paniculata* and *L. coromandelica* had significantly higher sapwood-specific hydraulic conductivity (*K*_S_) and leaf-specific hydraulic conductivity (*K*_L_) than *P. weinmanniifolia* (Figure 1, *P* < 0.001). However, no significant differences in *K*_S_ and *K*_L_ were found between *T. paniculata* and *L. coromandelica*.

**Figure 2:**
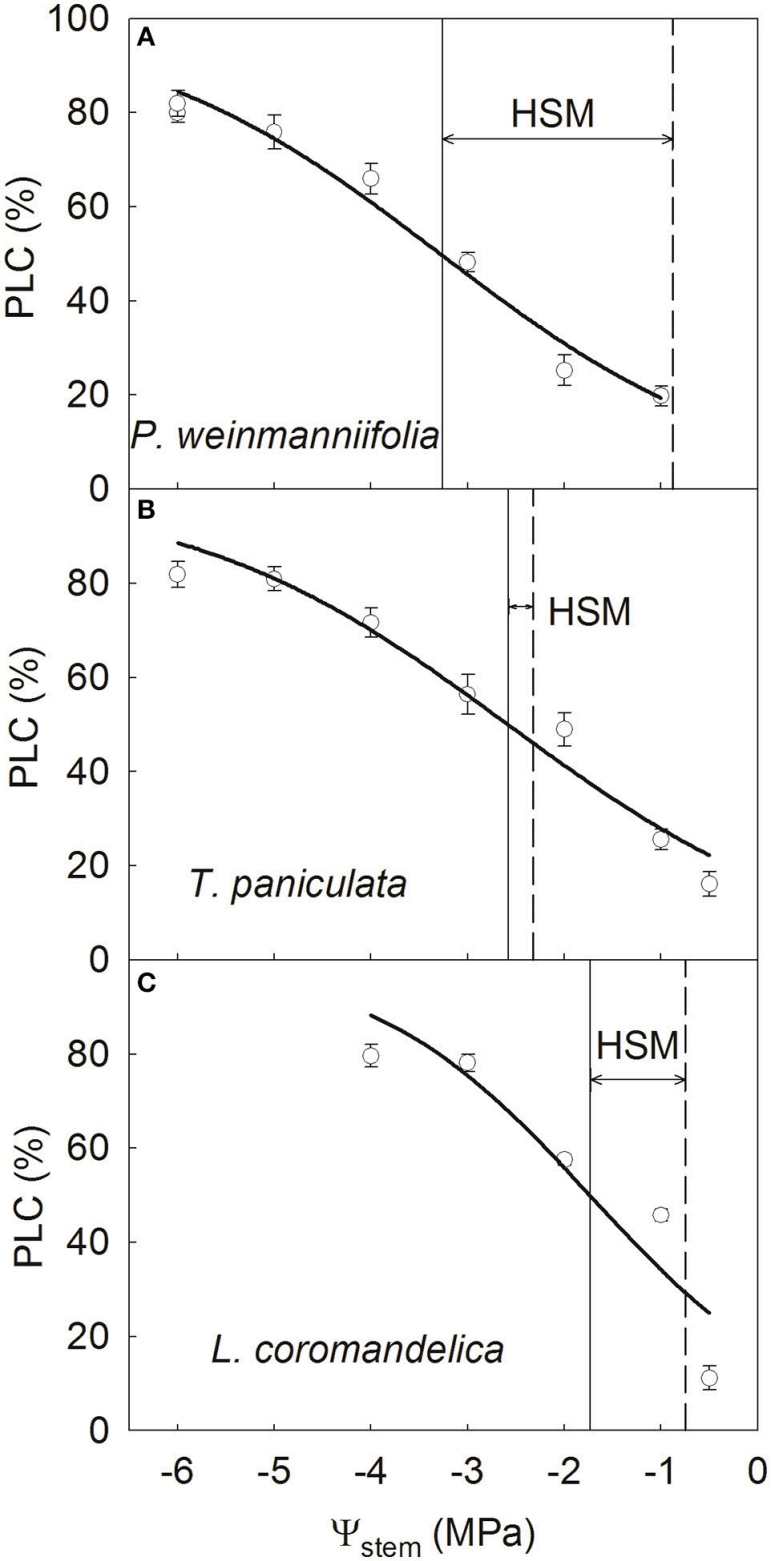
**Stem vulnerability curves of three Anacardiaceae tree species**. *P. weinmanniifolia* **(A)**, *T. paniculata* **(B)**, and *L. coromandelica* **(C)** Ψ_stem_, stem xylem water potential; PLC, percentage loss of sapwood hydraulic conductivity. The solid lines indicate the water potential at 50% loss of sapwood hydraulic conductivity, and the dashed lines indicate the minimum water potential in the stem. Values are means ± SE (*n* = 5).

Stem hydraulic vulnerability curves differed among the three species, with P50_stem_ ranging from −3.26 MPa in *P. weinmanniifolia* to −1.74 MPa in *L. coromandelica* (Figure 2). *P. weinmanniifolia* exhibited a wide HSM range in stems (2.38 MPa; Figure 2A); however, the HSM values in the stems reached their narrowest range (< 0.26 MPa) in *T. paniculata* (Figure 2B), which implies that the stems were at risk for drought-induced die-back. In addition, *L. coromandelica* displayed the most vulnerable leaves but a relatively wide HSM in the stems (1 MPa) (Figure 2C).

**Figure 3:**
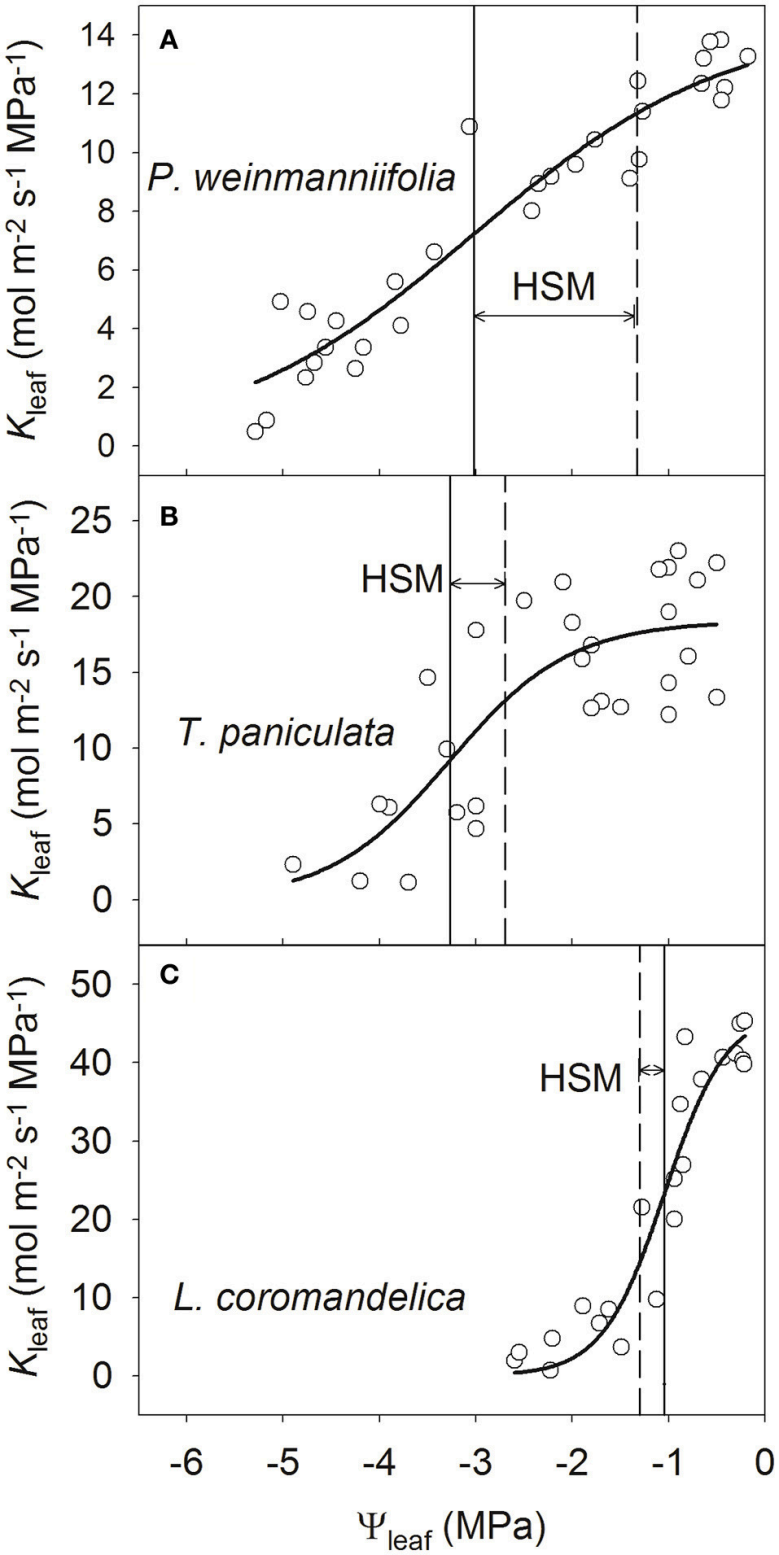
**Leaf vulnerability curves of three Anacardiaceae tree species**. *P. weinmanniifolia* **(A)**, *T. paniculata* **(B)**, and *L. coromandelica* **(C)** Ψ_leaf_, leaf water potential; *K*_leaf_, leaf hydraulic conductance. The solid lines indicate the water potential at 50% loss of *K*_leaf_, and the dashed lines indicate minimum water potential in the leaf.

The leaf vulnerability curves indicated that the leaves of *L. coromandelica* were the most vulnerable to drought-induced cavitation (P50_leaf_ = −1.05 MPa), but the value of P50_leaf_ was more negative in *T. paniculata* (−3.27 MPa) than in *P. weinmanniifolia* (−3.02 MPa) (Figure 3). Among the three species, *P. weinmanniifolia* exhibited the widest HSM in leaves (1.59 MPa) (Figure 3A); a negative value of HSM in leaves (−0.31 MPa) was found in *L. coromandelica* (Figure 3C). In addition, the HSM value in the leaves of *T. paniculata* exhibited a narrow range (0.57 MPa) by the late dry season (Figure 3B). These small HSMs indicate that the leaves were vulnerable to hydraulic dysfunction in two deciduous tree species as a result of increasing drought stress.

**Figure 4:**
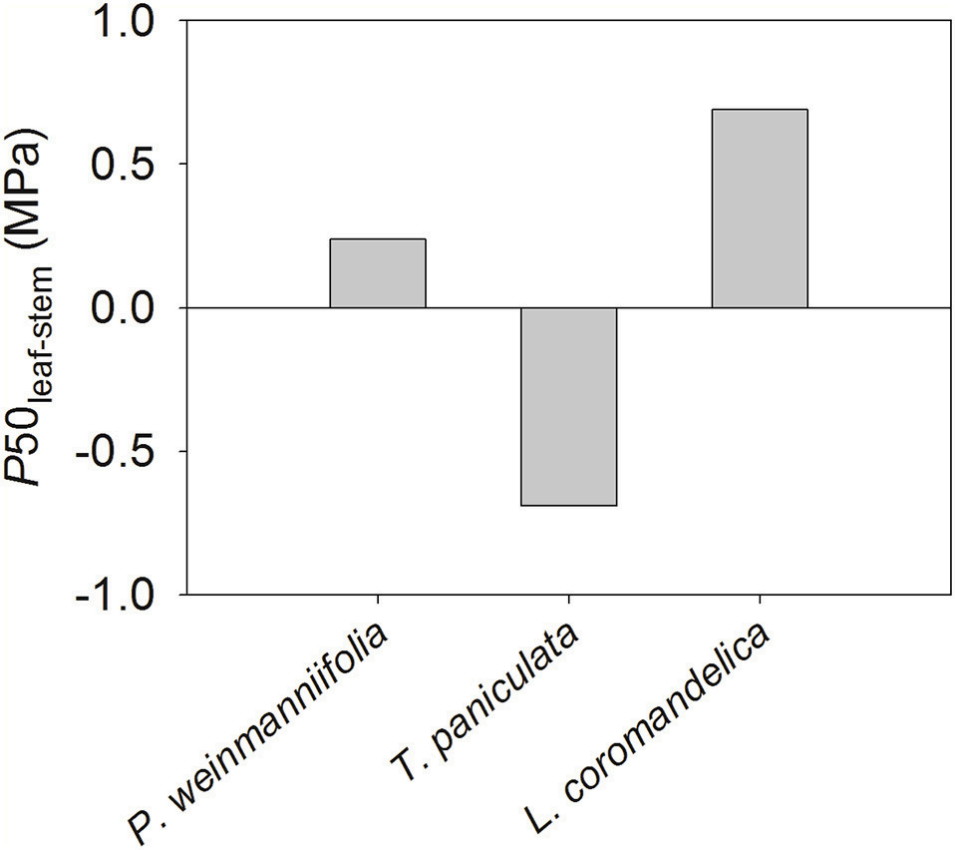
**Differences in water potential at 50% loss of hydraulic conductivity between the leaves and the terminal stems (P50_**leaf−stem**_) in three Anacardiaceae tree species**.

The difference in water potential at 50% loss of hydraulic conductivity between the leaves and the terminal stems (P50_leaf−stem_) was positive in *L. coromandelica* (0.69 MPa) and *P. weinmanniifolia* (0.24 MPa), but a negative value in *T. paniculata* (–0.69 MPa) (Figure 4).

### Leaf and stem hydraulic-related traits

*L. coromandelica* had the lowest LMA among the three studied species (Table 2, *P* < 0.001), but *P. weinmanniifolia* and *T. paniculata* exhibited non-significant differences in LMA (Table 2). *P. weinmanniifolia* and *T. paniculata* had significantly higher values of HV than *L. coromandelica* (*P* < 0.05 and *P* < 0.01, respectively). *P. weinmanniifolia* had the largest WD, the narrowest vessels, and the thickest vessel walls among the three species, whereas, *L. coromandelica* had the lowest WD, the widest vessels, and the thinnest vessel walls among the three species. *P. weinmanniifolia* exhibited the highest VD (104 no. mm^−2^), but *T. paniculata* and *L. coromandelica* had similar VD values (79 and 78 no. mm^−2^, respectively). In addition, the three species displayed non-significant differences in VGI.

**Table 2:** **Hydraulic-related leaf and stem traits of three Anacardiaceae tree species**.

**Traits**	**Abbreviation**	**Units**	***P. weinmanniifolia***	***T. paniculata***	***L. coromandelica***
Leaf mass per area	LMA	g m^−2^	135.5 ± 5.7a	123.1 ± 3.3a	66.9 ± 1.5b
Huber value	HV	mm^2^ m^−2^	1.74 ± 0.16a	1.83 ± 0.14a	1.31 ± 0.11b
Sapwood density	WD	g cm^−3^	0.67 ± 0.00a	0.54 ± 0.01b	0.34 ± 0.01c
Vessel diameter	D_h_	μm	38.1 ± 1.4a	60.4 ± 1.2b	69.3 ± 1.3c
Vessel wall thickness	VWT	μm	7.6 ± 0.2a	5.8 ± 0.3b	3.8 ± 0.2c
Vessel density	VD	no. mm^−2^	104 ± 10a	79 ± 9b	78 ± 8b
Vessel grouping index	VGI	–	3.01 ± 0.33a	2.43 ± 0.12a	2.44 ± 0.16a

*Values are means ± SE (n = 6). Different letters after means ± SE of hydraulic-related traits indicate significant differences among species at P < 0.05*.

## Discussion

### Hydraulic safety strategies differ across the three tree species

The difference between P50_leaf_ and P50_stem_ (P50_leaf−stem_) was positive in *P. weinmanniifolia* and *L. coromandelica* (Figure 4). This result supports the vulnerability segmentation hypothesis, which suggests that the leaves act as “safety valves” to protect hydraulic pathways from dysfunction (Tyree et al., 1993). In addition, *P. weinmanniifolia* and *L. coromandelica* maintained a relatively constant midday Ψ_leaf_, although predawn Ψ_leaf_ changed significantly during different seasons (Table 1). The patterns of seasonally constant midday Ψ_leaf_ indicate that these two tree species were isohydric according to the paradigm proposed by Franks et al. (2007). This isohydric behavior has been commonly found in savanna tree species (Bucci et al., 2003, 2005). Constant midday Ψ_leaf_ has been attributed to strong stomatal regulation of transpiration rate (Tardieu and Simonneau, 1998; Mcculloh and Meinzer, 2015).

By the early dry season, *g*_s_ decreased to 18.2% of the values in the rainy season with a 0.55 MPa decrease of predawn Ψ_leaf_ for *L. coromandelica* (Table 1). Strong stomatal regulation maintains a relatively wide HSM in stems of this species. In fact, this tree species possessed the most vulnerable stems and leaves, as demonstrated by its least negative P50_stem_ and P50_leaf_ among the three species (Figures 2C, 3C). The more vulnerable leaves may have greatly decreased *K*_leaf_ values under mild and modest drought, thereby inducing stomatal closure (Bucci et al., 2012; Liu et al., 2015). By regulating stomatal aperture, plants can control transpirational water loss and minimize fluctuations in xylem water potential (Franks et al., 2007). Even with the arrival of drought, Ψ_leaf_ decreased to a value below P50_leaf_ in *L. coromandelica*; this species thus exhibited a small negative HSM in leaves (Figure 3C) and it began to shed leaves. Therefore, its drought-avoidance strategy can maintain the hydraulic safety of the more carbon-costly stems by sacrificing cheaper and more vulnerable leaves (McDowell et al., 2008; Pivovaroff et al., 2014).

In contrast, both Ψ_leaf_ and Ψ_stem_ in *P. weinmanniifolia* were not affected significantly as a result of highly drought-resistant stems and leaves at the onset of drought (Table 1). Until peak drought, *g*_s_ decreased to 25.6% of the value in the rainy season with a 0.72 MPa decrease of predawn Ψ_leaf_ for *P. weinmanniifolia*, indicating strong stomatal regulation of the transpiration rate (Franks et al., 2007). *P. weinmanniifolia* maintained relatively wide HSM values both in stem (2.38 MPa) and leaf (1.69 MPa) under peak drought (Figures 2A, 3A), and this can prevent excessive embolisms in the long-distance water transport pathway (Meinzer et al., 2009) and help to prevent catastrophic hydraulic dysfunction during seasonal drought (Bucci et al., 2013; Liu et al., 2015). Thus, *P. weinmanniifolia* exhibited a hydraulic strategy of drought tolerance with strong stomatal regulation.

With respect to vulnerability segmentation, an opposite pattern was found in *T. paniculata*, a species with stems that are more vulnerable to cavitation than leaves (Figure 4), a pattern similar to some studies in Patagonian shrubs (Bucci et al., 2013) and many tree species in tropical rain forests (Zhu et al., 2016). The leaves in *T. paniculata* may not serve as “safety valves” to protect their stems against embolism. A few reports have indicated that species without vulnerability segmentation display effective hydraulic compensatory strategies such as a decline in leaf area and greater hydraulic efficiency (Bucci et al., 2012, 2013; Zhu et al., 2016). *T. paniculata* maintains a high *g*_s_ and transpiration rate in the early dry season (Table 1). During peak drought, the HSM in both stem and leaf decreased to a narrow range in *T. paniculata* (< 0.6 MPa, Figures 2B, 3B); however, this species still had a relatively high *g*_s_ (56.8% of the value in the rainy season; Table 1). This robust water use strategy may have resulted in excessive embolism via air seeding, and both stems and leaves were at risk of catastrophic hydraulic failure (Choat et al., 2012); consequently, some top shoots would die back (Kondoh et al., 2006). Indeed, a recent survey done in the Chinese savanna long-term monitoring plot at this site revealed that some top shoots died back in *T. paniculata* (S.B. Zhang, unpublished data). Rood et al. (2000) found that sacrificing some branches provided a drought adaptation for coping with drought stress in riparian cottonwoods, because transpirational loss was greatly reduced by dieback of some top branches. After sacrificing some top shoots and all of the leaves attached to these branches, *T. paniculata* sheds leaves to maintain the hydraulic safety of the whole plant until peak drought. Dieback of branches during peak seasonal drought can be seen as a segmentation strategy to protect the main stems and roots. Climate models project an increase in both the mean and extreme precipitation in the Indian summer monsoon in East, South, and Southeast Asia, however, there may be an increase in the interannual standard deviation of seasonal mean precipitation (Hijioka et al., 2014), thus more prolonged and severe seasonal drought. In this context, a small HSM in stems may result in substantial die-back of branches in *T. paniculata*, and even tree mortality at a regional scale.

### Hydraulic safety is related to leaf and stem structural traits

Previous studies suggested that the selection pressure induced by water deficit promoted differentiation in hydraulic traits among phenological groups (Markesteijn et al., 2011; Fu et al., 2012). Our results reveal that vulnerability to cavitation differs among co-occurring tree species with different leaf habits (Figures 2, 3), and these differences are related to leaf and stem structural traits (Table 2).

As LMA is one of the leading functional traits involved in plant growth and water use strategy (Poorter et al., 2009), it is closely associated with leaf hydraulic conductance and drought-resistance (Sack and Holbrook, 2006; Chen et al., 2009; Hao et al., 2011). LMA reflects the dry-mass cost to develop a new leaf (Wright et al., 2004). A lower LMA value is associated with a shorter leaf lifespan and a lower carbon-cost of deploying a new leaf (Coley, 1988). In this study, the leaves of *L. coromandelica* with the lowest LMA values had the highest *K*_leaf_ but were most vulnerable to cavitation among three tree species studied (Figure 3C). This result suggests a relatively lower carbon-cost of deploying a new leaf in *L. coromandelica* than in *P. weinmanniifolia* and *T. paniculata*. Hydraulic resistance from the leaves may account for 30–80% of the whole-plant resistance in the long-distance hydraulic pathway along the soil-plant-atmosphere continuum (Becker et al., 1999; Sack et al., 2002; Sack and Holbrook, 2006). Thus, the high LMA in *P. weinmanniifolia* and *T. paniculata* indicates that forming new leaves is a high carbon-cost investment in those species. Thus, it is important to maintain a wide HSM in the leaves in these two species, in order to tolerate seasonal drought.

Wood density integrates properties of the vessels and the matrix of fibers and thus is a key functional trait of sapwood hydraulic conductivity and cavitation resistance (Meinzer, 2003; Santiago et al., 2004; Markesteijn et al., 2011). In this study, conservative stem structural traits in *P. weinmanniifolia* (higher WD and VD, thicker VWT, narrower D_h_) promoted cavitation resistance (a more negative P50_stem_) and maintained a wide HSM in stems. These structural traits facilitated drought tolerance in the evergreen species. In contrast, wider vessels and lower WD contribute to higher sapwood hydraulic efficiency (higher *K*_S_ and *K*_L_) in two deciduous species, but with higher vulnerability to cavitation in the stems. Therefore, the significant differences in WD and vessel anatomical traits among the three tree species are directly related to the trade-off between hydraulic efficiency and safety, which is consistent with previous reports (Hacke et al., 2001, 2006; Onoda et al., 2010; Lens et al., 2011).

## Conclusions

Taken together, our results suggest that even sympatric tree species can exhibit divergent adaptive hydraulic safety strategies. More specifically, vulnerability segmentation is found in the evergreen *P. weinmanniifolia* and the winter-deciduous *L. coromandelica*. The winter-deciduous species exhibits a drought-avoidance strategy that maintains the hydraulic safety of the more carbon-costly stems by sacrificing the cheaper and more vulnerable leaves, while the evergreen species shows a hydraulic strategy of drought tolerance with strong stomatal regulation. In contrast, the drought-deciduous *T. paniculata* lacks vulnerability segmentation and sheds leaves at the cost of some top shoots during peak drought. In addition, the differences in leaf and stem hydraulic architecture among these species are closely related to their structural traits. Our results imply that these hydraulic properties of these tree species have co-evolved with their structural traits. Choat et al. (2012) reported that there is a small HSM range for angiosperm trees in global forests. More frequent and prolonged droughts are predicted due to ongoing global climate change, which may result in forest dieback at global scales (Allen and Breshears, 2007). Hydraulic safety of the whole plant can be maintained by sacrificing some distal portions of the shoots and leaves in deciduous tree species. Thus, it is important to distinguish differences related to leaf habits for the evaluation of the hydraulic safety of tree species and predict species distribution under climate change. Moreover, given the profound impact of HSMs on the growth and survival of tree species, possible links between hydraulic safety strategies and tree mortality need to be further investigated in different phenological groups at the landscape level.

## Author contributions

SZ and JZ designed experiments. SZ carried out experiments. SZ and KC wrote the manuscript.

## Funding

This study was funded by the National Key Research and Development Program of China (2016YFC0502102-05), the National Natural Science Foundation of China (31600479, 31570406, 31470470), and the CAS “Light of West China” Program to SZ.

### Conflict of interest statement

The authors declare that the research was conducted in the absence of any commercial or financial relationships that could be construed as a potential conflict of interest.

## References

[B1] Allen C. D. Breshears D. D. (2007). Climate-induced forest dieback as an emergent global phenomenon. Eos Trans. AGU. 88:504 10.1029/2007EO470008

[B2] Becker P. Tyree M. T. Tsuda M. (1999). Hydraulic conductances of angiosperms versus conifers: similar transport sufficiency at the whole-plant level. Tree Physiol. 19, 445–452. 10.1093/treephys/19.7.445 12651550

[B3] Brodribb T. J. Field T. S. (2000). Stem hydraulic supply is linked to leaf photosynthetic capacity: evidence from New Caledonian and Tasmanian rainforests. Plant Cell Environ. 23, 1381–1381. 10.1046/j.1365-3040.2000.00647.x

[B4] Brodribb T. J. Holbrook N. M. (2003a). Changes in leaf hydraulic conductance during leaf shedding in seasonally dry tropical forest. New Phytol. 158, 295–303. 10.1046/j.1469-8137.2003.00736.x

[B5] Brodribb T. J. Holbrook N. M. (2003b). Stomatal closure during leaf dehydration, correlation with other leaf physiological traits. Plant Physiol. 132, 2166–2173. 10.1104/pp.103.023879 12913171 PMC181300

[B6] Brodribb T. J. Holbrook N. M. (2004). Diurnal depression of leaf hydraulic conductance in a tropical tree species. Plant Cell Environ. 27, 820–827. 10.1111/j.1365-3040.2004.01188.x

[B7] Bucci S. J. Goldstein G. Meinzer F. C. Franco A. C. Campanello P. Scholz F. G. (2005). Mechanisms contributing to seasonal homeostasis of minimum leaf water potential and predawn disequilibrium between soil and plant water potential in Neotropical savanna trees. Trees Struct. Funct. 19, 296–304. 10.1007/s00468-004-0391-2

[B8] Bucci S. J. Scholz F. G. Campanello P. I. Montti L. Jimenez-Castillo M. Rockwell F. A.. (2012). Hydraulic differences along the water transport system of South American *Nothofagus* species: do leaves protect the stem functionality? Tree Physiol. 32, 880–893. 10.1093/treephys/tps054 22684354

[B9] Bucci S. J. Scholz F. G. Goldstein G. Meinzer F. C. Sternberg L. Da S. L. (2003). Dynamic changes in hydraulic conductivity in petioles of two savanna tree species: factors and mechanisms contributing to the refilling of embolized vessels. Plant Cell Environ. 26, 1633–1645. 10.1046/j.0140-7791.2003.01082.x

[B10] Bucci S. J. Scholz F. G. Peschiutta M. L. Arias N. S. Meinzer F. C. Goldstein G. (2013). The stem xylem of patagonian shrubs operates far from the point of catastrophic dysfunction and is additionally protected from drought-induced embolism by leaves and roots. Plant Cell Environ. 36, 2163–2174. 10.1111/pce.12126 23639077

[B11] Chen J. W. Cao K. F. (2015). A possible link between hydraulic properties and leaf habits in *Hevea brasiliensis*. Funct. Plant Biol. 42, 718–726. 10.1071/FP14294 32480715

[B12] Chen J. W. Zhang Q. Cao K. F. (2009). Inter-species variation of photosynthetic and xylem hydraulic traits in the deciduous and evergreen Euphorbiaceae tree species from a seasonally tropical forest in south-western China. Ecol. Res. 24, 65–73. 10.1007/s11284-008-0482-4

[B13] Choat B. Ball M. C. Luly J. G. Holtum J. A. M. (2005). Hydraulic architecture of deciduous and evergreen dry rainforest tree species from north-eastern Australia. Trees Struct. Funct. 19, 305–311. 10.1007/s00468-004-0392-1

[B14] Choat B. Jansen S. Brodribb T. J. Cochard H. Delzon S. Bhaskar R.. (2012). Global convergence in the vulnerability of forests to drought. Nature 491, 752–755. 10.1038/nature11688 23172141

[B15] Cochard H. Cruiziat P. Tyree M. T. (1992). Use of positive pressure to establish vulnerability curves: further support for the air-seeding hypothesis and implications for pressure-volume analysis. Plant Physiol. 100, 205–209. 10.1104/pp.100.1.205 16652947 PMC1075538

[B16] Coley P. D. (1988). Effects of plant growth rate and leaf lifetime on the amount and type of anti-herbivore defense. Oecologia 74, 531–536. 10.1007/BF00380050 28311759

[B17] Delzon S. Cochard H. (2014). Recent advances in tree hydraulics highlight the ecological significance of the hydraulic safety margin. New Phytol. 203, 355–358. 10.1111/nph.12798 24661229

[B18] Franks P. J. (2006). Higher rates of leaf gas exchange are associated with higher leaf hydrodynamic pressure gradients. Plant Cell Environ. 29, 584–592. 10.1111/j.1365-3040.2005.01434.x 17080609

[B19] Franks P. J. Drake P. L. Froend R. H. (2007). Anisohydric but isohydrodynamic: seasonally constant plant water potential gradient explained by a stomatal control mechanism incorporating variable plant hydraulic conductance. Plant Cell Environ. 30, 19–30. 10.1111/j.1365-3040.2006.01600.x 17177873

[B20] Fu P. L. Jiang Y. J. Wang A. Y. Brodribb T. J. Zhang J. L. Zhu S. D.. (2012). Stem hydraulic traits and leaf water-stress tolerance are co-ordinated with the leaf phenology of angiosperm trees in an asian tropical dry karst forest. Ann. Bot. 110, 189–199. 10.1093/aob/mcs092 22585930 PMC3380589

[B21] Hacke U. G. Jansen S. (2009). Embolism resistance of three boreal conifer species varies with pit structure. New Phytol. 182, 675–686. 10.1111/j.1469-8137.2009.02783.x 19309447

[B22] Hacke U. G. Sperry J. S. Pockman W. T. Davis S. D. McCulloh K. A. (2001). Trends in wood density and structure are linked to prevention of xylem implosion by negative pressure. Oecologia 126, 457–461. 10.1007/s004420100628 28547229

[B23] Hacke U. G. Sperry J. S. Wheeler J. K. Castro L. (2006). Scaling of angiosperm xylem structure with safety and efficiency. Tree Physiol. 26, 1689–1701. 10.1093/treephys/26.6.689 16510385

[B24] Hao G. Y. Goldstein G. Sack L. Holbrook N. M. Liu Z. H. Wang A. Y.. (2011). Ecology of hemiepiphytism in fig species is based on evolutionary correlation of hydraulics and carbon economy. Ecology 92, 2117–2130. 10.1890/11-0269.1 22164836

[B25] Hijioka Y. Lin E Pereira J. J. Corlett R. T. Cui X. Insarov G. E. (2014). ‘Asia’ in Climate Change 2014: Impacts, Adaptation, and Vulnerability. Part B: Regional Aspects, in Contribution of Working Group II to the Fifth Assessment Report of the Intergovernmental Panel on Climate Change. eds Barros V. R. Field C. B. Dokken D. J. Mastrandrea M. D. Mach K. J. Bilir T. E. (Cambridge, UK; New York, NY: Cambridge University Press, Cambridge), 1327–1370.

[B26] Holbrook N. M. Whitbeck J. L. Mooney H. A. (1995). Drought responses of Neotropical dry forest trees, in Seasonally Dry Tropical Forests, eds Bullock S. H. Mooney H. A. Medina E. (Cambridge, UK: Cambridge University Press), 243–270. 10.1017/CBO9780511753398.010

[B27] Ishida A. Harayama H. Yazaki K. Ladpala P. Sasrisang A. Kaewpakasit K. (2010). Seasonal variations in hydraulic properties of deciduous and evergreen trees in monsoonal dry forests of Thailand. Tree Physiol. 30, 935–945. 10.1093/treephys/tpq025 20581012

[B28] Jin Z. Z. Ou X. K. (2000). Vegetations in the Hot and Dry Valleys along the Yuanjiang, Nujiang, Jinshajiang, and Lanchangjiang Rivers. Kunming: Yunnan University Press (in Chinese).

[B29] Johnson D. M. McCulloh K. A. Woodruff D. R. Meinzer F. C. (2012). Hydraulic safety margins and embolism reversal in stems and leaves: why are conifers and angiosperms so different? Plant Sci. 195, 48–53. 10.1016/j.plantsci.2012.06.010 22920998

[B30] Johnson D. M. Woodruff D. R. Mcculloh K. A. Meinzer F. C. (2009). Leaf hydraulic conductance, measured in situ, declines and recovers daily: leaf hydraulics, water potential and stomatal conductance in four temperate and three tropical tree species. Tree Physiol. 29, 879–887. 10.1093/treephys/tpp031 19429900

[B31] Johnson D. M. Wortemann R. McCulloh K. A. Jordan-Meille L. Ward E. Warren J. M.. (2016). A test of the hydraulic vulnerability segmentation hypothesis in angiosperm and conifer tree species. Tree Physiol. 36, 983–993. 10.1093/treephys/tpw031 27146334

[B32] Kondoh S. Yahata H. Nakashizuka T. Kondoh M. (2006). Interspecific variation in vessel size, growth and drought tolerance of broadleaved trees in semi-arid regions of Kenya. Tree Physiol. 26, 899–904. 10.1093/treephys/26.7.899 16585035

[B33] Lens F. Sperry J. S. Christman M. A. Choat B. Rabaey D. Jansen S. (2011). Testing hypotheses that link wood anatomy to cavitation resistance and hydraulic conductivity in the genus *Acer*. New Phytol. 190, 709–723. 10.1111/j.1469-8137.2010.03518.x 21054413

[B34] Liu Y. Y. Song J. Wang M. Li N. Niu C. Y. Hao G. Y. (2015). Coordination of xylem hydraulics and stomatal regulation in keeping the integrity of xylem water transport in shoots of two compound-leaved tree species. Tree Physiol. 35, 1333–1342. 10.1093/treephys/tpv061 26209618

[B35] Markesteijn L. Poorter L. Paz H. Sack L. Bongers F. (2011). Ecological differentiation in xylem cavitation resistance is associated with stem and leaf structural traits. Plant Cell Environ. 34:137–148. 10.1111/j.1365-3040.2010.02231.x 20946587

[B36] Mcculloh A. K. Meinzer C. F. (2015). Further evidence that some plants can lose and regain hydraulic function daily. Tree Physiol. 35, 691–693. 10.1093/treephys/tpv066 26163489

[B37] McDowell N. Pockman W. T. Allen C. D. Breshears D. D. Cobb N. Kolb T.. (2008). Mechanisms of plant survival and mortality during drought: why do some plants survive while others succumb to drought? New Phytol. 178, 719–739. 10.1111/j.1469-8137.2008.02436.x 18422905

[B38] Meinzer F. C. (2003). Functional convergence in plant responses to the environment. Oecologia 134, 1–11. 10.1007/s00442-002-1088-0 12647172

[B39] Meinzer F. C. Johnson D. M. Lachenbruch B. McCulloh K. A. Woodruff D. R. (2009). Xylem hydraulic safety margins in woody plants: coordination of stomatal control of xylem tension with hydraulic capacitance. Funct. Ecol. 23, 922–930. 10.1111/j.1365-2435.2009.01577.x

[B40] Nolf M. Creek D. Duursma R. Holtum J. Mayr S. Choat B. (2015). Stem and leaf hydraulic properties are finely coordinated in three tropical rain forest tree species. Plant Cell Environ. 38, 2652–2661. 10.1111/pce.12581 26032606

[B41] Onoda Y. Richards A. E. Westoby M. (2010). The relationship between stem biomechanics and wood density is modified by rainfall in 32 Australian woody plant species. New Phytol. 185, 493–501. 10.1111/j.1469-8137.2009.03088.x 19925557

[B42] Pammenter N. W. Vander Willigen C. (1998). A mathematical and statistical analysis of the curves illustrating vulnerability of xylem to cavitation. Tree Physiol. 18, 589–593. 10.1093/treephys/18.8-9.589 12651346

[B43] Pivovaroff A. L. Sack L. Santiago L. S. (2014). Coordination of stem and leaf hydraulic conductance in southern California shrubs: a test of the hydraulic segmentation hypothesis. New Phytol. 203, 842–850. 10.1111/nph.12850 24860955

[B44] Poorter H. N. U. Poorter L. Wright I. J. Villar R. (2009). Causes and consequences of variation in leaf mass per area (LMA): a meta-analysis. New Phytol. 192, 565–588. 10.1111/j.1469-8137.2009.02830.x 19434804

[B45] Poorter L. Markesteijn L. (2008). Seedling traits determine drought tolerance of tropical tree species. Biotropica 40, 321–331. 10.1111/j.1744-7429.2007.00380.x

[B46] Rood S. B. Patiño S. Coombs K. Tyree M. T. (2000). Branch sacrifice: cavitation-associated drought adaptation of riparian cottonwoods. Trees 14, 248–257. 10.1007/s004680050010

[B47] Sack L. Holbrook N. M. (2006). Leaf hydraulics. Annu. Rev. Plant Biol. 57, 361–381. 10.1146/annurev.arplant.56.032604.144141 16669766

[B48] Sack L. Melcher P. J. Zwieniecki M. A. Holbrook N. M. (2002). The hydraulic conductance of the angiosperm leaf lamina: a comparison of three measurement methods. J. Exp. Bot. 53, 2177–2184. 10.1093/jxb/erf069 12379784

[B49] Santiago L. S. Goldstein G. Meinzer F. C. Fisher J. B. Machado K. Woodruff D.. (2004). Leaf photosynthetic traits scale with hydraulic conductivity and wood density in Panamanian forest canopy trees. Oecologia 140, 543–550. 10.1007/s00442-004-1624-1 15232729

[B50] Sperry J. S. Donnelly J. R. Tyree M. T. (1988). A method for measuring hydraulic conductivity and embolism in xylem. Plant Cell Environ. 11, 35–40. 10.1111/j.1365-3040.1988.tb01774.x

[B51] Sperry J. S. Tyree M. T. (1988). Mechanism of water stress-induced xylem embolism. Plant Physiol. 88, 581–587. 10.1104/pp.88.3.581 16666352 PMC1055628

[B52] Tardieu F. Simonneau T. (1998). Variability among species of stomatal control under fluctuating soil water status and evaporative demand: modelling isohydric and anisohydric behaviours. J. Exp. Bot. 49, 419–432. 10.1093/jxb/49.Special_Issue.419

[B53] Tyree M. T. Cochard H. Cruiziat P. Sinclair B. Ameglio T. (1993). Drought-induced leaf shedding in walnut: evidence for vulnerability segmentation. Plant Cell Environ. 16, 879–882. 10.1111/j.1365-3040.1993.tb00511.x

[B54] Tyree M. T. Davis S. D. Cochard H. (1994). Biophysical perspectives of xylem evolution: is there a tradeoff of hydraulic efficiency for vulnerability to dysfunction? IAWA J. 15, 335–360. 10.1163/22941932-90001369

[B55] Tyree M. T. Zimmermann M. H. (2002). Xylem Structure and the Ascent of Sap. New York, NY: Springer-Verlag 10.1007/978-3-662-04931-0

[B56] Wheeler J. K. Sperry J. S. Hacke U. G. Hoang N. (2005). Inter-vessel pitting and cavitation in woody Rosaceae and other vesselled plants: a basis for a safety versus efficiency trade-off in xylem transport. Plant Cell Environ. 28, 800–812. 10.1111/j.1365-3040.2005.01330.x

[B57] Wright I. J. Reich P. B. Westoby M. Ackerly D. D. Baruch Z. Bongers F.. (2004). The worldwide leaf economics spectrum. Nature 428, 821–827. 10.1038/nature02403 15103368

[B58] Zhang J. L. Zhu J. J. Cao K. F. (2007). Seasonal variation in photosynthesis in six woody species with different leaf phenology in a valley savanna in southwestern China. Trees Struct. Funct. 21, 631–643. 10.1007/s00468-007-0156-9

[B59] Zhang Y. J. Meinzer F. C. Qi J. H. Goldstein G. Cao K. F. (2013). Midday stomatal conductance is more related to stem rather than leaf water status in subtropical deciduous and evergreen broadleaf trees. Plant Cell Environ. 36, 149–158. 10.1111/j.1365-3040.2012.02563.x 22715809

[B60] Zhu S. D. Liu H. Xu Q. Y. Cao K. F. Ye Q. (2016). Are leaves more vulnerable to cavitation than branches? Funct. Ecol. 30, 1740–1744. 10.1111/1365-2435.12656

[B61] Zimmermann M. H. (1978). Hydraulic architecture of some diffuse-porous trees. Can. J. Bot. 56, 2286–2295. 10.1139/b78-274

