# Supplementary material for: Divergent Hydraulic Safety Strategies in Three Co-occurring Anacardiaceae Tree Species in a Chinese Savanna
Source: Front Plant Sci. 2017 Jan 18;7:2075. doi: 10.3389/fpls.2016.02075 (PMC5241295; doi:10.3389/fpls.2016.02075)
Supplement: Supplementary file 1 [file DataSheet1.docx]

## Supplementary Figure 1


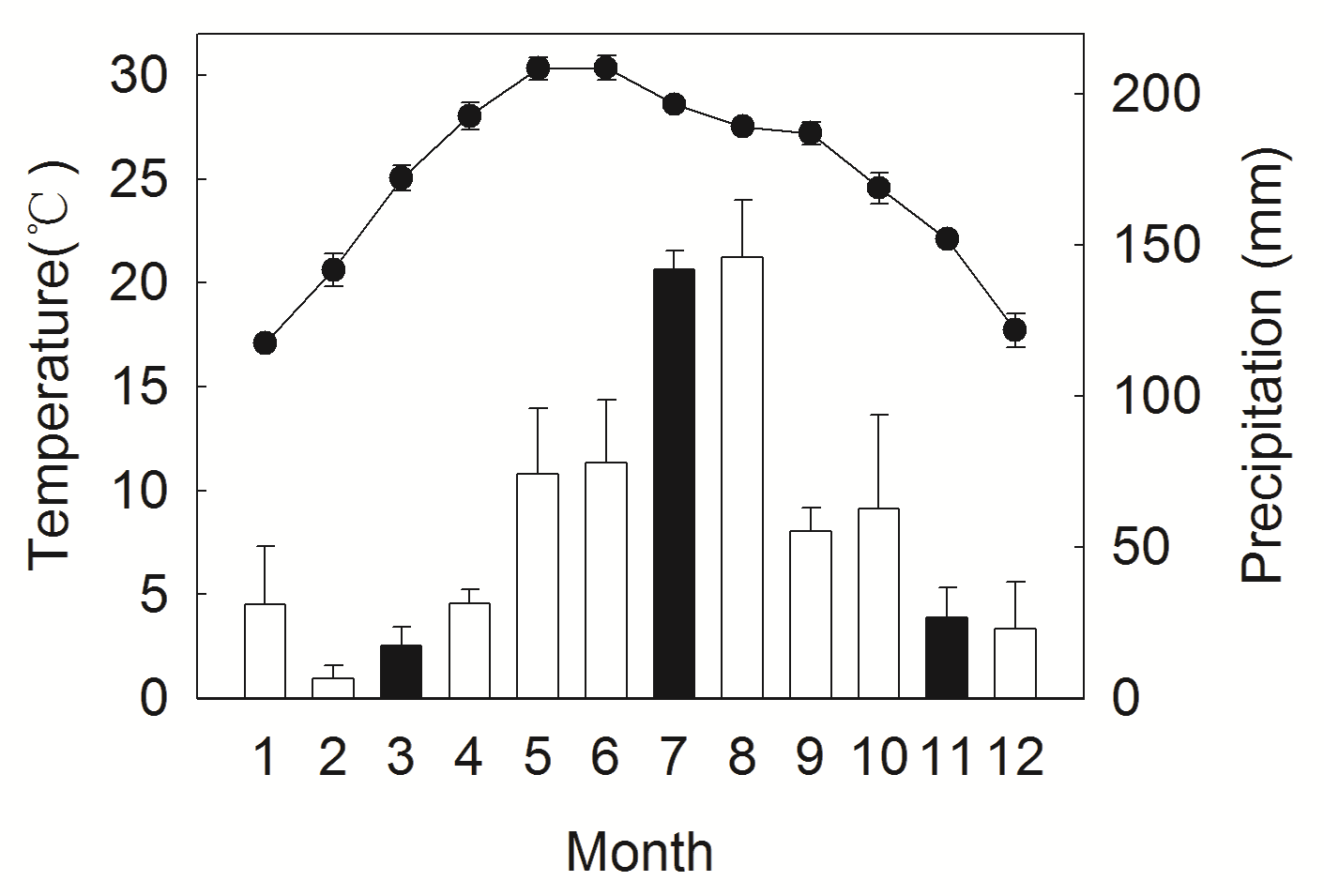


**Supplementary Figure 1.** Monthly average air temperature (black circles) and monthly precipitation (vertical bars) at the study site. Black bars indicate the months when seasonal samples were collected. The dry season lasts from November to the next April, so the July, November, and March samples were collected during the rainy season, the early dry season, and the late dry season, respectively. Values are the means of meteorological data from 2012–2015.

## Supplementary Table 1. Characteristics of the three Anacardiaceae tree species studied. Values are the means of five sample individuals per species.

| **Species** | **Leaf phenology** | **Height** | **DBH** |
| --- | --- | --- | --- |
| *Pistacia weinmanniifolia* | evergreen | 6.0±0.6 m | 15.8±1.8 cm |
| *Terminthia paniculata* | drought-deciduous | 5.4±0.4 m | 11.2±0.9 cm |
| *Lannea coromandelica* | winter-deciduous | 6.4±0.3 m | 15.7±1.6 cm |
